# An Autopsy Case of Fulminant Amebic Colitis in a Patient with a History of Rheumatoid Arthritis

**DOI:** 10.1155/2016/8470867

**Published:** 2016-06-12

**Authors:** Naoko Kawabe, Fuyuki Sato, Miho Nagasawa, Masako Nakanishi, Yasuteru Muragaki

**Affiliations:** Department of Pathology, Wakayama Medical University School of Medicine, Wakayama 641-8509, Japan

## Abstract

Generally, amebic colitis is localized around the mucosal membrane and often accompanied by diarrhea and abdominal pain. We describe a patient with a history of rheumatoid arthritis who had received prolonged steroid therapy. The patient complained of breathing difficulties because of rheumatoid lung disease. Although the patient was given antibacterial agent, the symptoms did not improve until death. We did an autopsy and found that he had fulminant amebic colitis, although the patient was not previously examined. Histochemical analysis revealed severe inflammation and full-thickness necrosis of the colon by ameba, suggesting the involvement of ameba in the progression of the overall condition.

## 1. Introduction

Rheumatoid arthritis (RA) patients have high risk of infections by cytomegalovirus, pneumocystis jirovecii, and nontuberculous mycobacterium [1]. Hashimoto and Matsui reported infectious lesions: 52% of respiratory system, 11% of kidney and urinary organs, 10% of gastrointestinal system, and 8% of skin and soft tissues [1]. It has been reported that treating autoimmune hepatitis patient with prolonged steroid induced amebic encephalitis [2]. In addition, prolonged steroid treatment and further steroid administration of 10 mg/day induced the progression of amebic colitis and pulmonary infections [3–6].

## 2. Case Presentation

A 74-year-old man with a history of RA has received prolonged steroid therapy. He was treated with steroid for at least 1 week during the hospitalization, but we could not obtain how long he had been treated with steroid therapy before the hospitalization of university. Several months before his death, the patient was suspected to have respiratory distress and deterioration of rheumatic symptoms and was diagnosed with interstitial pneumonia by chest CT. Rheumatic symptoms were improved by steroid therapy, but fever and inflammatory reaction continued. Therefore, he was treated with internal antibacterial agent. Since his symptoms continued even after those treatments, he was hospitalized.

**Figure 1:**
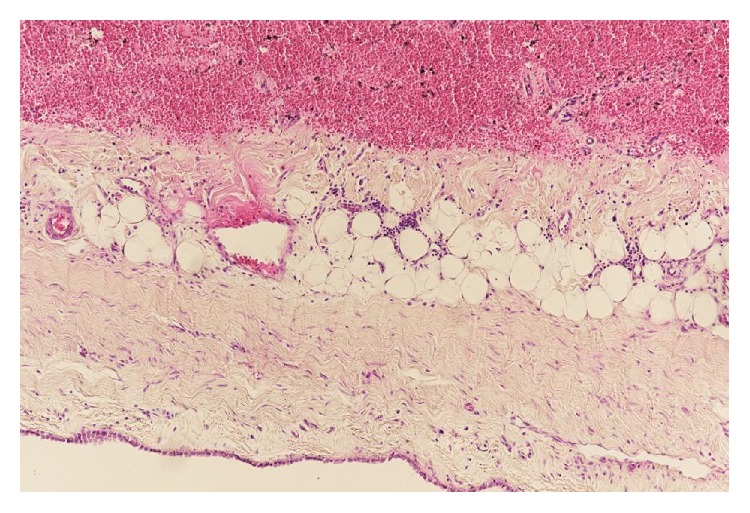
Histopathology (×100). Fibrosis and hemorrhage in pleura.

**Figure 2:**
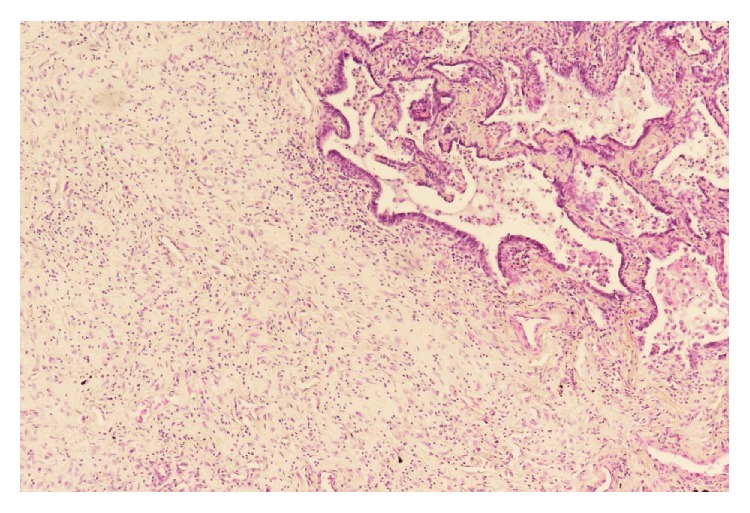
Histopathology (×100). Interstitial pneumonia.

**Figure 3:**
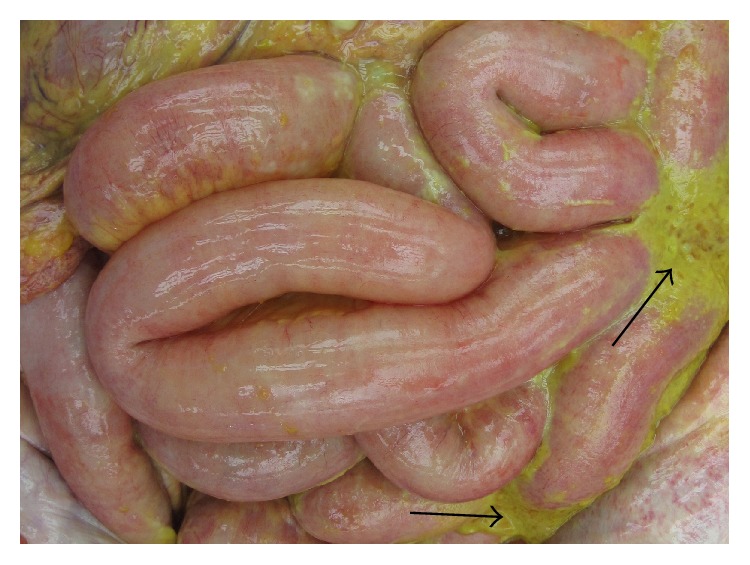
Pus in the colon.

**Figure 4:**
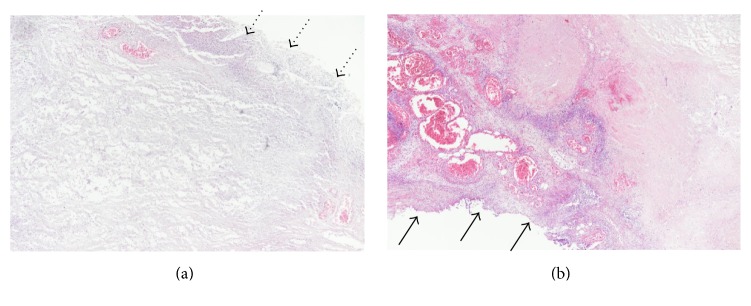
Histopathology (×100). Necrosis from mucous membrane (a) to serous membrane (b). Dotted arrow shows mucous membrane. Black arrow shows serous membrane.

**Figure 5:**
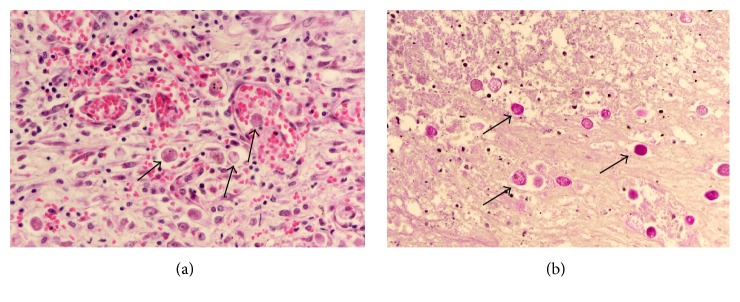
Histopathology (×400). Infiltration of ameba in the colon. Arrow shows ameba. (a) Hematoxylin and eosin staining. (b) Periodic acid-Schiff staining.

Laboratory examinations on admission showed white blood cells at 21.8 × 10^9^/L, C-reactive protein (CRP) at 23.51 mg/dL, albumin (Alb) at 1.8 g/dL, Krebs von den Lungen-6 (KL-6) at 486 U/mL, surfactant protein A (SP-A) at 27.4 ng/mL, surfactant protein D (SP-D) at 40.7 ng/mL, procalcitonin (PCT) at 0.17 ng/mL, and creatinine (Cre) at 0.68 mg/dL. On the same day, chest CT showed ground-glass opacity and reticular shadow in the left middle and inferior lobes as well as the left inferior lingular segment. Because his procalcitonin elevated, he was treated with piperacillin and tazobactam for infectious diseases. For the hypoalbuminemia, he was treated with a supplementation of albumin. However, his fever did not improve, suggesting progression of rheumatic lung disease. Therefore, he was given prednisolone at 60 mg/day. On hospital day six, he had onset of diarrhea and bloody stool, which might be caused by the side effect of the antibacterial drug. On day eight, because his procalcitonin level was even higher than on admission day, steroid was stopped. On day ten, he had recurrent fever and increased white blood cells and CRP and was given 1 g of prednisolone for three hours. On day 13, his Cre increased, while urine volume decreased. He was prescribed predonisolone 60 mg/day. On day 15, his CRP was at 29.15 mg/dL. On that day, examination of the blood showed white blood cells at 26.9 × 10^9^/L and procalcitonin at 64.59 ng/mL. He was clinically diagnosed as sepsis. He lost consciousness and progression of respiratory distress, resulting in cardiopulmonary arrest and death. We performed autopsy on that day. The pleura was replaced due to bleeding and fibrous thickening (Figure 1). In addition, alveolar wall was severely thick and inflamed, suggesting compatibility with the finding of interstitial pneumonia (Figure 2). He had peritonitis with marked pus in the colon (Figure 3) as well as full-thickness necrotic tissues of the colon from the mucosal (Figure 4(a)) to serous membrane (Figure 4(b)). Severe infiltration of ameba was detected in microvessel of colon (Figure 5(a)). We performed PAS staining for positive detection of ameba in colon (Figure 5(b)). Severe neutrophil infiltration was observed in liver, spleen, colon, stomach, pancreas, heart, and lungs, suggesting compatibility with the findings of sepsis (data not shown).

## 3. Discussion

We pathologically found severe fibrosis of alveolar wall and pleura. These findings are compatible with rheumatoid lung disease, suggesting that he had been suffering from the disease for a prolonged period. There was also the possibility of amebic infection.

Amebiasis is caused by the protozoan* Entamoeba histolytica*, which dominantly affects the colon and liver. The main route of transmission depends on ingestion of amebae from food or water contaminated with feces [7, 8]. A majority of infected patients remain asymptomatic. However, acute fulminant amebic colitis can be fatal [9]. Although amebiasis is not epidemic in Japan, the prevalence is increasing due to sexually transmitted diseases [10, 11]. In particular, the increasing prevalence is largely attributed to more people travelling to affected geographical locations, as well as a rise in the prevalence of HIV and male homosexuality [10, 11]. In this case, the patient was HIV-negative, and we could not determine if he had travelled to an affected area.

Therefore, the etiology of the amebic infection remained unknown.

Generally, amebic colitis affects the mucosa and its surrounding [5, 6, 12, 13]. However, few reports had shown that steroid treatment for inflammatory bowel disease induced amebic colitis treated by steroid colonic perforation with full-thickness necrosis [8]. We found amebic colitis with full-thickness necrosis and perforation as well as amebic infection in the liver and fat tissue around the pancreas. In addition, we observed vascular infiltration of ameba in the colon and liver, suggesting that the amebic infection has spread to other organs via the vascular system. RA and prolonged and high dosed steroid treatment may have induced the amebic infection, which could be the reasons for the patient's severe infection. The cause of death may be due to bacterial sepsis.

In conclusion, severe amebic infection may induce transmural necrosis and perforation of colon. RA and prolonged and high dosed steroid treatment may be important risk factors for fulminant amebic colitis.
